# IKKα regulates human keratinocyte migration through surveillance of the redox environment

**DOI:** 10.1242/jcs.197343

**Published:** 2017-03-01

**Authors:** Thomas S. Lisse, Sandra Rieger

**Affiliations:** 1 Davis Center for Regenerative Biology and Medicine, MDI Biological Laboratory, 159 Old Bar Harbor Road, Salisbury Cove, ME 04672, USA; 2 The Jackson Laboratory, Bar Harbor, ME 04609, USA

**Keywords:** Wound repair, Wound healing, Keratinocytes, Migration, Reactive oxygen species, Hydrogen peroxide, H_2_O_2_, IKKα, Sulfenylation, EGF, Nuclear de-repression, HEK001, Oxidation

## Abstract

Although the functions of H_2_O_2_ in epidermal wound repair are conserved throughout evolution, the underlying signaling mechanisms are largely unknown. In this study we used human keratinocytes (HEK001) to investigate H_2_O_2_-dependent wound repair mechanisms. Scratch wounding led to H_2_O_2_ production in two or three cell layers at the wound margin within ∼30 min and subsequent cysteine modification of proteins via sulfenylation. Intriguingly, exogenous H_2_O_2_ treatment resulted in preferential sulfenylation of keratinocytes that adopted a migratory phenotype and detached from neighboring cells, suggesting that one of the primary functions of H_2_O_2_ is to stimulate signaling factors involved in cell migration. Based on previous findings that revealed epidermal growth factor receptor (EGFR) involvement in H_2_O_2_-dependent cell migration, we analyzed oxidation of a candidate upstream target, the inhibitor of κB kinase α (IKKα; encoded by *CHUK*), as a mechanism of action. We show that IKKα is sulfenylated at a conserved cysteine residue in the kinase domain, which correlates with de-repression of EGF promoter activity and increased EGF expression. Thus, this indicates that IKKα promotes migration through dynamic interactions with the EGF promoter depending on the redox state within cells.

## Abstract (precis)

**Summary:** This study provides a newly identified mechanism by which H_2_O_2_-dependent oxidation of the inhibitor of κB kinase α and de-repression of epidermal growth factor promoter activity stimulates keratinocyte migration.

## INTRODUCTION

During the early tissue formation stage of the cutaneous wound repair process, re-lining of wounds by basal keratinocytes restores an intact epidermal barrier (Arwert et al., 2012; Rieger et al., 2014). This step involves dynamic physiological and molecular changes to initiate migration over the provisional matrix and proliferation of keratinocytes from the surrounding epidermis (Usui et al., 2005; Watt, 2002). A number of soluble factors modulate wound repair (Gault et al., 2014; Gurtner et al., 2008; Niethammer, 2014), including the small reactive oxygen species (ROS) hydrogen peroxide (H_2_O_2_) (Loo et al., 2011; Niethammer et al., 2009). The H_2_O_2_ signal transduction cascade is evolutionarily conserved and acts via receptors, protein kinases, structural components and downstream transcription factor-dependent, post-translational and genomic mechanisms (Gough and Cotter, 2011). H_2_O_2_ is an immediate wound attractant signal for *Drosophila* hemocytes (Moreira et al., 2010), and H_2_O_2_ gradients within the wounded epithelium are crucial for phagocytic cell migration (Niethammer et al., 2009; Tauzin et al., 2014) through direct oxidation of the Src-family kinase member Lyn (Yoo et al., 2011). H_2_O_2_ also promotes the regrowth of peripheral sensory axons and their reinnervation of healing skin (Rieger and Sagasti, 2011), and it is essential for tail regeneration of *Xenopus* tadpoles (Love et al., 2013). Addition of exogenous H_2_O_2_ to wounds accelerates wound closure in mice (Loo et al., 2012; Roy et al., 2006) and in keratinocyte culture injury models (Loo and Halliwell, 2012; Loo et al., 2011; Pan et al., 2011). Thus, H_2_O_2_ appears to be critical for wound repair; yet the molecular understanding of this physiological function remains elusive.

At low concentrations, H_2_O_2_ functions as a second messenger whereby it oxidizes cysteine thiols with low pKa in signaling proteins, which are often found in catalytic domains of signaling enzymes (Claiborne et al., 1999). Oxidation of cysteine thiols stimulates the formation of sulfenic acid (sulfenylation), a highly unstable metabolite that can rapidly convert into other metabolites, such as sulfinic and sulfonic acid, or nitrosothiol (Leonard et al., 2009). A common metabolic process in which sulfenic acid participates is the promotion of intramolecular disulfide bond formation. This alters protein conformation and modulates the activation or inactivation of enzymes (Leonard and Carroll, 2011; Stone and Yang, 2006; Truong and Carroll, 2012a). As a consequence, H_2_O_2_ can modulate phosphorylation cascades within cells, often by activating kinases and deactivating phosphatases (Claiborne et al., 1999; Gough and Cotter, 2011).

The inhibitor of κB kinase α (IKKα) (encoded by *CHUK*) represents a candidate for oxidative regulation of H_2_O_2_ during migration. First, IKKα is structurally related to its homolog IKKβ (encoded by *IKBKB*), which has been shown to be oxidized by arsenite at a conserved cysteine residue in the kinase domain (Kapahi et al., 2000). Second, we have recently shown that IKKα is essential for keratinocyte migration *in vitro* and acts downstream of H_2_O_2_ (Lisse et al., 2016). Third, IKKα has a non-conventional role in repressing *EGF* and *HBEGF* promoter activity to control epithelial differentiation and prevent tumor formation during skin homeostasis (Hu et al., 1999, 2001; Liu et al., 2008; Marinari et al., 2008; Sil et al., 2004). For example, ablation of IKKα leads to increased keratinocyte proliferation and impairment of terminal differentiation (Hu et al., 2001; Sil et al., 2004). In addition, IKKα plays an important role in human cancers, as its dysfunction has been reported in squamous cell carcinomas of the skin (Liu et al., 2006). Studies have focused primarily on the role of IKKα during epidermal terminal differentiation, but whether it is involved in other cellular activities, such as migration, has however not been examined. In addition, it is unclear whether the keratinocyte-specific molecular and biological functions of IKKα can be modulated by oxidation, similar to IKKβ. Given our previous findings implicating IKKα in migration, we speculated that IKKα might serve as an intracellular surveilling protein to promote either migration or differentiation depending on the redox environment. We hypothesized that oxidation of IKKα can promote the de-repression of *EGF* promoter activity, which may stimulate migration via EGFR signaling.

## RESULTS

### H_2_O_2_ activates human keratinocyte migration

To investigate cell-autonomous molecular mechanisms regulating H_2_O_2_-induced keratinocyte migration, we utilized a human epidermal keratinocyte line (HEK001) (Sugerman and Bigby, 2000). We first validated that HEK001 cellular responses were similar to those in primary keratinocytes. We found that these cells differentiated after treatment with high (2 mM) Ca^2+^ concentrations (Fig. S1A). *PCNA* (proliferation marker), *KRT14* (basal keratinocyte marker) and *AXIN2*, an inhibitor of the Wnt pathway, were expressed during the first 7 days *in vitro* (DIV). *KRT10* (early differentiation marker) and *IVL* (late differentiation marker) showed a lower level of expression by 24 h, which gradually increased until day 7 *in vitro*. These markers seemed to increase further when cells were treated with high Ca^2+^ concentrations. The increase in PCNA and KRT14 expression ≥7 DIV suggests that this cell line shows aberrant differentiation characteristics. Regardless, 7-DIV cultures, when treated with high Ca^2+^ (2 mM), showed increased stratification (Fig. S1B) and aggregation of terminally differentiated lipid-forming keratinocytes when assessed with Crystal Violet staining (Fig. S1C). 7-DIV cultures, moreover, failed to close scratch wounds even after 72 h (Fig. S1D), which correlated with reduced H_2_O_2_ formation at the scratch wound, as assessed with pentafluorobenzenesulfonyl-fluorescein (HPF) (Fig. S1E,F). These findings show that migration is initiated in undifferentiated keratinocytes that resemble more closely a progenitor-like population (at 2 DIV), consistent with *in vivo* characteristics of basal keratinocytes.

**Fig. 1.**
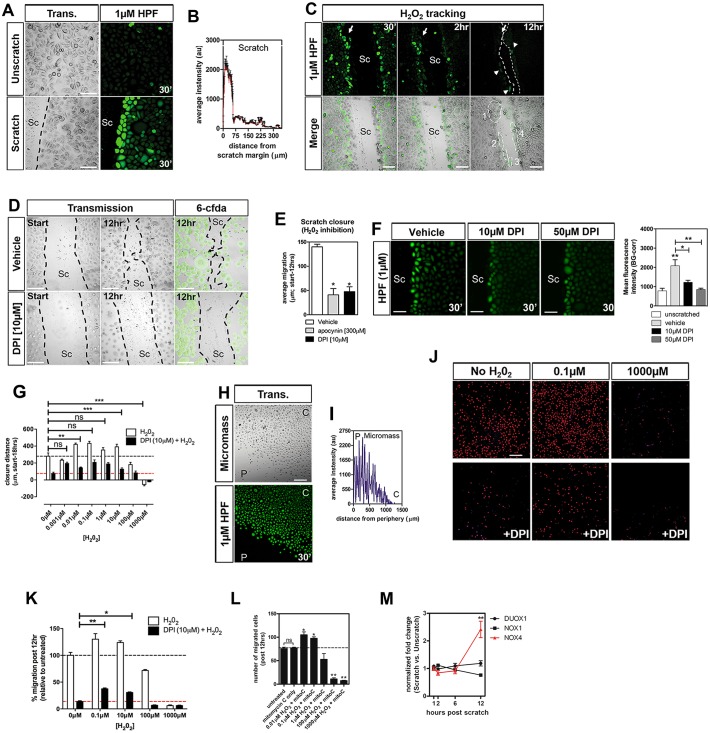
**Endogenous and exogenous H_2_O_2_ activates HEK001 migration.** (A) Rapid H_2_O_2_ formation at the scratch (Sc) wound margin detected with HPF. Trans., transmission. (B) Quantification of H_2_O_2_ production as shown in A. (C) H_2_O_2_ tracking during scratch repair shows diminished HPF signal shortly before scratch closure. (D) DPI inhibition of keratinocyte migration after scratch with limited cytotoxicity, assessed using the cell viability dye 6-CFDA. (E) Treatment of cells with apocynin and DPI impairs scratch wound repair. (F) Diminished H_2_O_2_ levels at the scratch margin after DPI treatment. (G) Accelerated scratch closure upon treatment with exogenous H_2_O_2_ when compared with endogenous H_2_O_2_-dependent scratch closure (depicted by black dotted line). Addition of exogenous H_2_O_2_ to DPI-treated cells promotes scratch closure at rates similar to those of endogenous closure. DPI treatment reduces wound closure (red dotted line depicts baseline for comparisons with DPI+H_2_O_2_; black dotted line depicts the baseline for comparisons with H_2_O_2_.). (H) HPF staining in a micromass assay shows strongest fluorescence at the outer edges. ‘C’, confluent region; ‘P’, periphery. (I) Quantification of fluorescence intensity as shown in H. (J) Transwell migration assay performed with untreated cells and after treatment with H_2_O_2_. Migration is absent with DPI treatment but rescued upon supplementation of DPI-treated cells with H_2_O_2_. High H_2_O_2_ levels block migration. (K) Quantification of experiments shown in J. (L) Transwell migration assay in the presence of mitomycin C in the presence of 0.1 and 1 µM H_2_O_2_ shows increased migration, independent of proliferation. (M) *DUOX1*, *NOX1* and *NOX4* qPCR in unscratched and scratched HEK001 cells. SC (scratch). Scale bars: 100 µm. One-way ANOVA and Tukey's multiple comparison post hoc test were used. Significance: *P<0.05, **P<0.01, ***P<0.001 (*n*≥3-5 cell culture experiments). 30ʹ, 30 minutes after scratching.

Using *in vitro* scratch wound assays prior to day 7 *in vitro*, we observed rapid formation of H_2_O_2_ (∼30 min) after single-pulse labeling with HPF, with strongest signal formation in two or three cell layers at the scratch margin (Fig. 1A,B). For our studies, characterization of ROS signal-to-noise ratios showed that 1 µM but not 4 µM was critical to detect differential H_2_O_2_ levels, whereas higher concentrations resulted in toxicity (Fig. S2A-C). Migration onset occurred around ∼6 h and closure was typically seen between 12 and 18 h, which was batch-dependent (Fig. 1C). Closure coincided with downregulation of H_2_O_2_ in cells at the scratch margin. Migration was blocked by treatment with 10 µM of the general ROS inhibitor diphenyleneiodonium (DPI) (Fig. 1D left panel), but this was not caused by cellular toxicity, as cells were viable when assessed with 6-carboxyfluorescein diacetate (6-CFDA) (Fig. 1D, right panel). Also, the NADPH oxidase inhibitor apocynin blocked scratch repair (Fig. 1E), confirming our results. To validate that H_2_O_2_ levels were reduced upon inhibitor treatment, we assessed cells that had been pre-treated with DPI and HPF for 30 min before scratching, resulting in a significant reduction of H_2_O_2_ at the scratch margin (Fig. 1F). Thus, DPI does not decrease cell viability (Fig. 1D) but impairs migration (Fig. 1D,E). We next analyzed whether addition of exogenous H_2_O_2_ at concentrations ranging from 0.001-100 µM enhanced scratch closure. While low concentrations increased scratch repair, high H_2_O_2_ levels were inhibitory (Fig. 1G). We further performed micromass assays to test whether keratinocytes at the periphery of the micromass foci, which we assumed to be less differentiated than those in the center (Bedal et al., 2014; Stott et al., 1998), generated H_2_O_2_ similar to scratch margin cells. This showed elevated H_2_O_2_ levels at the outer edges compared with confluent regions where less H_2_O_2_/HPF signal was measured (Fig. 1H,I). We further utilized human primary keratinocytes to assess H_2_O_2_ production upon scratching and found a similar response as in HEK001 cells (Fig. S2D). Quantification of the migration distance after H_2_O_2_ treatment using 0.1 and 100 µM H_2_O_2_ showed increased migration at low levels but not high levels, which blocked migration (Fig. S2E). These findings suggest a concentration-dependent role for H_2_O_2_ in keratinocyte migration.

To dissect out the specific role of H_2_O_2_ (as opposed to other ROS) during scratch closure, we co-administered DPI and exogenous H_2_O_2_. We first assessed the time until scratch closure and found that DPI alone decreased closure within 18 h by 73%, whereas low H_2_O_2_ concentrations restored closure to near endogenous rates (Fig. 1G). To monitor bona fide migration, we utilized Transwell chambers and observed a similar increase in migration with addition of low H_2_O_2_ concentrations (Fig. 1J,K). Low-level (0.1 µM) H_2_O_2_ significantly promoted migration in the presence of DPI, but migration was reduced in DPI-treated cells and those treated with 100 and 1000 µM H_2_O_2_. We also performed Transwell assays after addition of the proliferation inhibitor mitomycin C using various concentrations of H_2_O_2_ to see whether proliferation accounts for the observed increase in cell number (Fig. 1L). This confirmed that the observed increase was related to bona fide migration. Thus, the presence of low H_2_O_2_ concentrations specifically induces keratinocyte migration. Finally, to assess specific NADPH oxidases involved in H_2_O_2_ production, we monitored *NOX1*, *NOX4* and *DUOX1* transcription using quantitative PCR (qPCR). This revealed that *NOX4*, but not *DUOX1* and *NOX1*, was significantly upregulated after scratch wounding at 12 h post scratch while remaining low in unscratched cells (Fig. 1M). Taken together, low concentrations of H_2_O_2_ promote migration of HEK001 keratinocytes, which leads to delayed upregulation of *NOX4* mRNA.

### H_2_O_2_-dependent signaling factors that are important for keratinocyte migration

We previously performed RNAseq and Ingenuity^®^ Upstream Regulator analyses to identify genome-wide transcriptional networks mediated by H_2_O_2_ and showed four major upstream networks with notable cutaneous associations (i.e. involving H_2_O_2_, EGF, FOXO1 and IKKα) following treatment of zebrafish with H_2_O_2_ (Lisse et al., 2016). Further sub-categorization of each network to define enriched pathways within them (Fig. S3) showed genes in each of these networks that functionally cluster into overlapping categories, including cell migration, defense response and wound repair. Genes involved in migration and which were differentially regulated in our data set included *FOXO1*, *IGFBP-1*, *HMOX1*, *HSPA1L* and *ITGB4*. All of those showed a dose-dependent increase in gene expression following H_2_O_2_ treatment of HEK001 cells (Lisse et al., 2016).

**Fig. 2.**
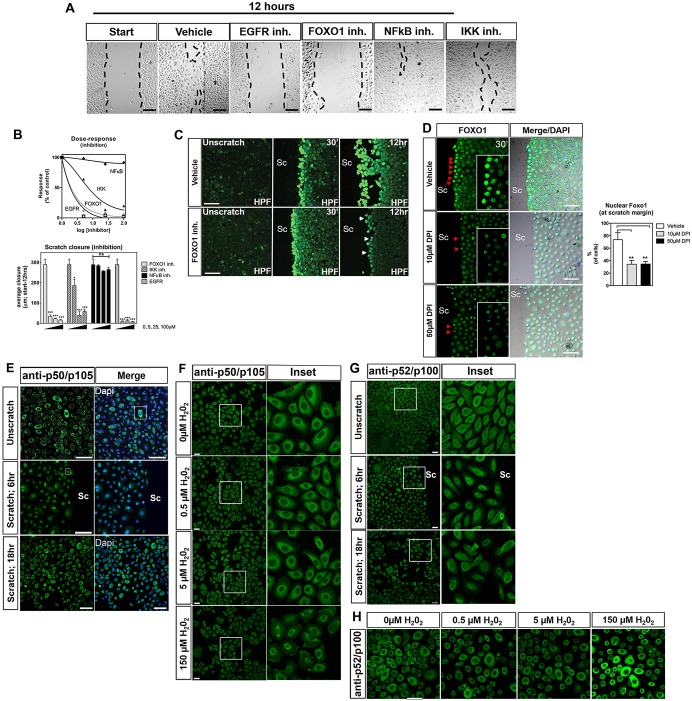
**Scratch wound assay defines candidates for oxidation-dependent repair.** (A) Effects of inhibitors on HEK001 scratch closure (5 µM sets shown). Inh., inhibitor. (B) Dose response of inhibitors measured using the scratch closure distance between the two edges of the wound. (C) HPF signals at the scratch margin in the absence and presence of FOXO1 inhibitor. SC, scratch. Time after scratching is shown in the top right. 30ʹ, 30 minutes. (D) Rapid nuclear localization of FOXO1 at the scratch margin of vehicle controls. Pre-treatment with DPI decreases FOXO1 staining at the scratch. Inset shows magnification of cells marked with red arrowheads. (E) Nuclear NF-κB1 (p50 and p105; p50/p105) is noticeable at the wound margin at 6 h post scratching but is cytoplasmic following wound closure at ∼18 h, similar to in unscratched (unscratch) cells. (F) Predominantly cytoplasmic NF-κB1 p50/p105 staining was found in untreated cells and following H_2_O_2_ treatment for 2 h. (G) Non-canonical NF-κB2 (p52 and p100; p52/p100) immunofluorescence following scratching shows predominantly cytoplasmic staining. (H) p52/p100 immunofluorescence reveals sustained cytoplasmic localization after H_2_O_2_ treatment for 2 h. High H_2_O_2_ concentrations lead to increased expression of NF-κB2. Scale bars: 100 µm (A-E,H); 50 µm (F,G). Two-way ANOVA and Bonferroni's multiple comparison post hoc tests were used. Significance: *P<0.05, ***P<0.001, ns: not significant (*n*≥3-5 cell culture experiments).

To investigate these networks further in H_2_O_2_-dependent keratinocyte migration, we pharmacologically blocked the function of EGFR, FOXO1 and IKKα–NF-κB. EGFR has a demonstrated role in migration and metastasis (Jones and Rappoport, 2014), and can be modulated through either direct oxidation (Paulsen et al., 2012) or phosphorylation (Loo et al., 2011). As expected, EGFR inhibition completely blocked injury-induced scratch repair (Fig. 2A,B). We next analyzed FOXO1 involvement in H_2_O_2_-dependent migration. Consistently, we observed an impairment of injury-induced cell migration after FOXO1 inhibition (Fig. 2A,B), but H_2_O_2_ production at the scratch margin was unaffected 30 min after scratching in FOXO1-inhibited HEK001 cells (Fig. 2C), suggesting an upstream regulatory function of H_2_O_2_. We did, however, observe a decrease in H_2_O_2_ levels at the unclosed scratch margin after 12 h of inhibitor treatment compared to controls. FOXO activity is tightly controlled by post-translational modifications, which mediate FOXO nuclear localization and degradation. We therefore assessed its subcellular localization after scratching and in the presence of DPI. Immunofluorescence studies showed rapid FOXO1 nuclear localization in keratinocytes at the scratch margin, which was attenuated by DPI (Fig. 2D). These results suggest that H_2_O_2_ regulates FOXO1 activity and nuclear localization/function during early wound repair. FOXO1 seems to modulate H_2_O_2_ levels at later stages, consistent with previous findings showing that it controls oxidative stress levels during wound re-epithelialization (Ponugoti et al., 2013).

We next assessed the role of IKKα in H_2_O_2_-dependent migration. Given that IKKα is best known for its regulation of the nuclear factor κB (NF-κB) pathway, which is implicated in oxidative stress responses (Gloire et al. 2006), we first determined if NF-κB was activated. Pharmacological inhibition of canonical NF-κB signaling by blocking RelA (p65) nuclear translocation (Shin et al., 2004) failed to impair scratch closure (Fig. 2A,B). We did, however, observe transient NF-κB1 p50 and p105 (two cleavage products encoded by *NFKB1*) nuclear localization at 6 h following scratch wounding (Fig. 2E). H_2_O_2_ treatment however did not promote nuclear localization (Fig. 2F), suggesting alternative functions in scratch margin cells, possibly induced by the oxidant-dependent phosphoinositide 3-kinase (PI-3K) ‘survival’ pathway (Sonoda et al., 1999). Also staining of NF-κB2 p52 and p100 (two cleavage products encoded by *NFKB2*) was predominantly cytoplasmic upon scratch wounding and H_2_O_2_ treatment (Fig. 2G,H). Thus, H_2_O_2_ does not appear to regulate NF-κB signaling during scratch wound repair.

**Fig. 3.**
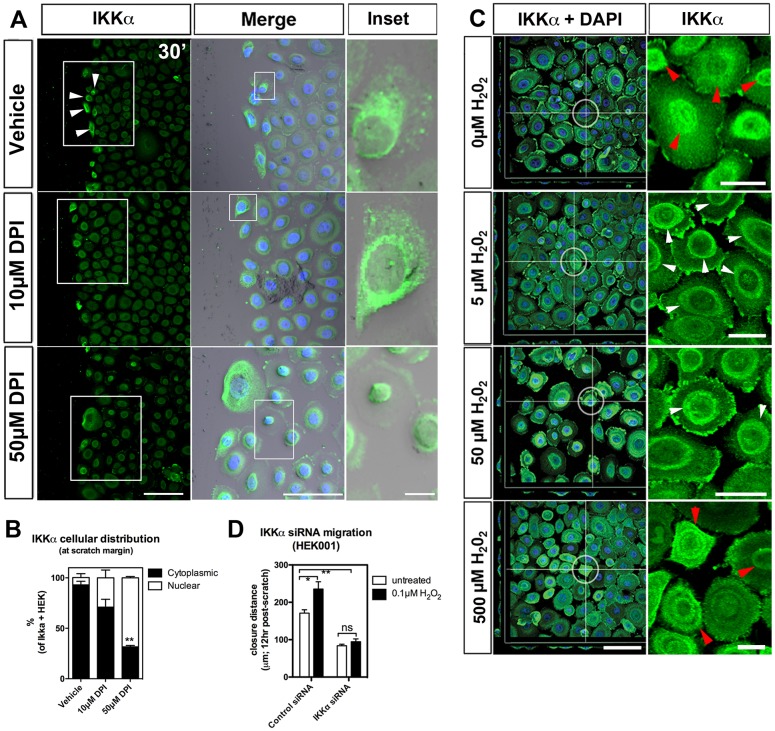
**H_2_O_2_-dependent regulation of IKKα during scratch repair.** (A) Rapid IKKα cytoplasmic and peri-nuclear localization (white arrowheads) at the wound margin 30 min (30ʹ) post-scratch. DPI treatment increases nuclear localization within the scratch area (see higher magnification insets). Arrowheads, demarcate IKKα-expressing cells at the scratch margin. Scale bars: 100 µm (left panels), 10 µm (insets). (B) Quantification of IKKα subcellular distribution from analyses shown in A. (C) IKKα localization within different compartments (measured after 2 h). Right panel shows higher magnifications. White arrowheads point to cytoplasmic perinuclear accumulation of IKKα at low H_2_O_2_ levels. Red arrowheads point to the nuclear accumulation and diffuse cytoplasmic staining of IKKα in untreated cells and after treatment with high H_2_O_2_ concentrations (500 µM). Scale bars: 50 µm (left panel), 10 µm (insets). (D) siRNA knockdown of IKKα prevents H_2_O_2_-induced scratch closure. Data are mean±s.e.m. Two-way ANOVA and Bonferroni's multiple comparison post hoc tests were utilized. Significance: *P<0.05, **P<0.01, ns: not significant (*n*≥3-5 cell culture experiments).

Given the absence of NF-κB involvement and our findings that IKKα is a predicted target of H_2_O_2_, we wanted to determine whether IKKα plays an NF-κB-independent role in scratch repair. Using the IKKα and IKKβ inhibitory compound, Wedelolactone, we found that scratch wound repair was impaired only when using high inhibitor concentrations of 25 and 50 µM (Fig. 2A,B). To further investigate IKKα functions, we assessed its (sub)cellular localization using immunofluorescence staining. We observed its predominant expression in keratinocytes that were adjacent to the scratch margin, where it was localized in the cytoplasm and perinuclear regions (Fig. 3A,B). Nuclear localization was however promoted with DPI (Fig. 3A,B). In addition, nuclear IKKα was also predominant after treatment with 2 mM Ca^2+^ to promote differentiation (Fig. S4A), and in cells that had been cultured for prolonged periods (7 DIV) following treatment with DPI (Fig. S4B) and after EGF withdrawal (Fig. S4C). Following H_2_O_2_ treatment of unscratched keratinocytes, staining was predominant in the perinuclear regions but nuclear or diffuse in untreated cells (Fig. 3C). At a high H_2_O_2_ concentration (500 µM), we found IKKα staining to be more diffuse in the cytoplasm and occasionally nuclear (Fig. 3C), suggesting that IKKα localization is regulated by H_2_O_2_ in a concentration-dependent manner. To further establish that IKKα is involved in H_2_O_2_-dependent repair, we assessed scratch closure with and without exogenous H_2_O_2_ treatment using siRNA against IKKα. IKKα mRNA and protein was specifically and significantly knocked down (>60%) following siRNA transfection (Fig. S4D-F). This correlated with the absence of cell migration following scratching (Fig. S4G). In the presence of endogenous and exogenous H_2_O_2_, control-siRNA-transfected keratinocytes showed closure rates comparable to those of untransfected cells, whereas IKKα knockdown impaired scratch closure under both conditions (Fig. 3D). Overall, these findings indicate that IKKα is regulated by H_2_O_2_ and essential for scratch wound repair.

### Post-translational cysteine oxidation of IKKα is involved in keratinocyte migration

**Fig. 4.**
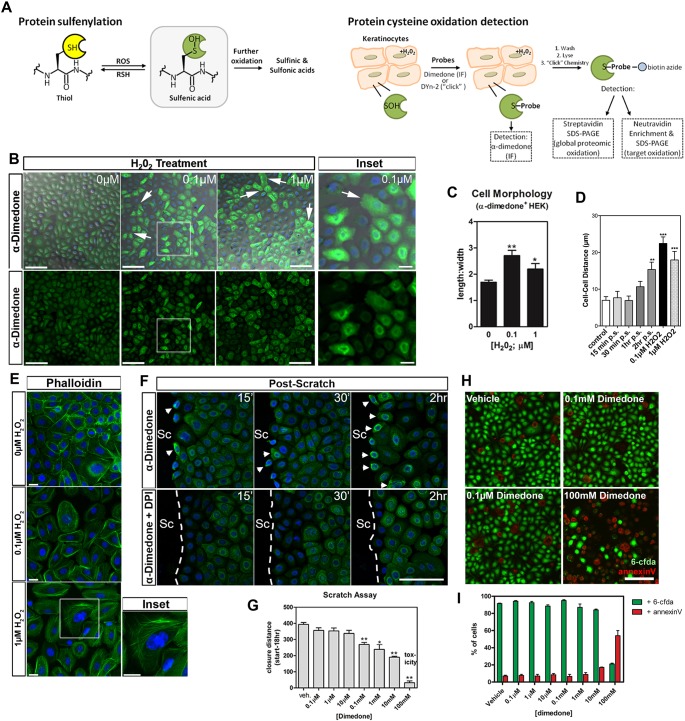
**Protein sulfenylation in HEK001 cells.** (A) Scheme describing dimedone- and DYn-2-labeling of cysteine-sulfenic acid (S-OH)-modified proteins. (B) Low-level treatment with H_2_O_2_ for 2 h induces sulfenylation in a subpopulation of cells with a migratory phenotype, showing lamellipodia formation (arrows, insets show magnifications). Scale bars: 100 µm (left panel), 10 µm (inset). (C) Sulfenylated cells display increased length:width ratios compared with non-sulfenylated cells. (D) Quantification of cell–cell distance between elongated, pro-migratory and neighboring cells. p.s., post scratch. (E) Phalloidin–AlexaFluor647 (green) staining shows increased stress fiber formation upon 0.1 and 1 µM H_2_O_2_ treatment. Scale bars: 20 µm. Blue, Hoechst 33342. (F) Cytoplasmic/peri-nuclear protein oxidation was found predominately in scratch margin cells (arrowheads), but sulfenylation is largely absent in scratched keratinocytes treated with DPI. Scale bar: 100 µm. Sc, scratch. Time after scratching is shown in the top right. 15ʹ, 15 min. (G) Dimedone treatment impairs scratch wound repair. Veh., vehicle. (H,I) Cell viability and death assessment using 6-CFDA and Annexin-V staining, respectively. One-way ANOVA and Tukey's multiple comparisons test were performed for statistical analyses. Data are mean±s.e.m. Scale bar: 100 µm.

Having shown the importance of IKKα during H_2_O_2_-dependent scratch repair, we next asked whether IKKα is directly oxidized by H_2_O_2_ at a conserved cysteine residue in the kinase domain, as has been shown for the structurally related kinase, IKKβ (Kapahi et al., 2000). We first determined global protein oxidation following 2 h H_2_O_2_ treatment and upon scratch wounding using the oxidation (sulfenylation)-selective compound, dimedone, followed by immunofluorescence staining for sulfenic acid (S-OH)–dimedone complexes (Paulsen et al., 2012; Truong, 2012a) (Fig. 4A). First we analyzed unscratched keratinocytes that had been treated with exogenous H_2_O_2_ (0.1 µM) as control. Interestingly, this revealed cysteine oxidation specifically in cells that appeared to have adopted a migratory phenotype, as indicated by the formation of lamellipodia at the leading edge (Fig. 4B). Also the length:width ratio of sulfenylated cells (Fig. 4C) increased, indicating a migratory fate (Rieger et al., 2009) consistent with cell detachment from neighboring cells (Fig. 4D). Phalloidin staining further revealed the formation of stress fibers in cells following low H_2_O_2_ treatment, which was uncommon in untreated cells (Fig. 4E). These findings suggest that H_2_O_2_ either selectively diffuses into cells to induce protein oxidation and migration, or it is degraded in a subpopulation of keratinocytes that do not become migratory. We next assessed scratch-dependent sulfenylation, which was predominant in the cytoplasm and perinuclear regions of cells at the wound margin (Fig. 4F, top row). The localization of sulfenylated proteins was comparable to that of the H_2_O_2_-dependent localization of IKKα (Fig. 3A,C), indicating that IKKα may be a sulfenylation target. Intriguingly, sulfenylation was most prominent after ∼2 h, indicating that this process might be highly regulated. To validate sulfenylation specificity, we further treated keratinocytes with DPI and then performed dimedone staining, which showed diminished signals at the scratch margin (Fig. 4F, bottom row). To test whether sulfenylation is required for scratch repair, we treated keratinocytes with dimedone, which specifically and irreversibly reacts with sulfenylated cysteine residues (Seo and Carroll, 2011). Addition of dimedone at various concentrations before scratch wounding impaired wound closure in a dose-dependent manner (Fig. 4G). We found that the highest dimedone concentration (100 mM) promoted cellular toxicity, as assessed with 6-CFDA, and apoptosis, as assessed with Annexin-V staining (Fig. 4H,I).

**Fig. 5.**
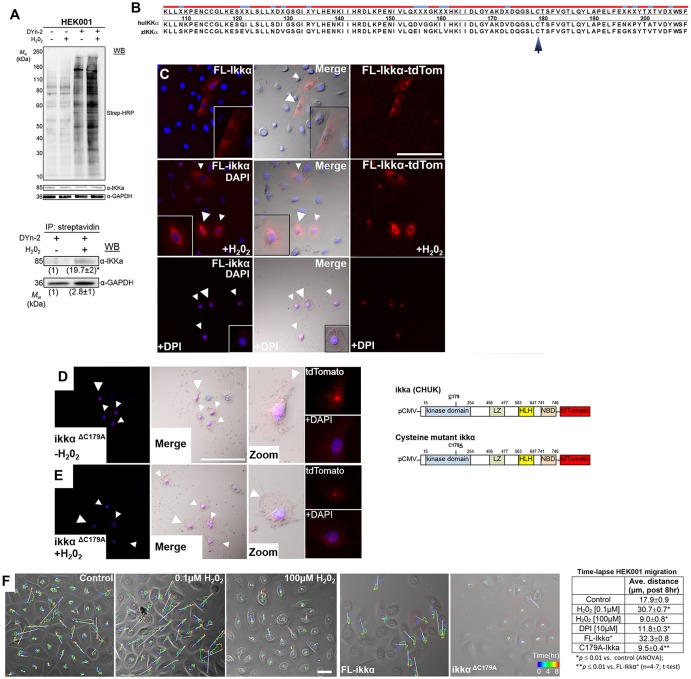
**IKKα sulfenylation promotes keratinocyte migration.** (A) Upper blot: DYn-2 probe-loaded keratinocytes were biotinylated using ‘click chemistry’ with a biotinylated azide probe. Sulfenylated proteins were probed with streptavidin (Strep)–HRP (upper panel). IKKα is detected in all fractions (with or without DYn-2 and H_2_O_2_). Lower blot: H_2_O_2_ oxidizes IKKα, assessed after purification of the biotinylated fraction by streptavidin pull-down and probing with anti-IKKα antibody. GAPDH serves as positive control for successful pull down of oxidized/sulfenylated proteins given its redox function (Truong, 2012a). IP, immunoprecipitation; WB, western blot. Values under the blots depict fold-changes from baseline controls. (B) Human (Hu) and zebrafish (z) IKKα kinase domain sequence comparison shows high conservation (∼70%), including zebrafish residue cysteine 179 (arrow). Red, conserved residues; blue, non-conserved residues. (C) IKKα construct scheme for HEK001 *in vitro* transfection studies are shown to the right of panel D. Full-Length (FL) Ikkα–tdTomato in cytoplasmic perinuclear regions with and without H_2_O_2_ (white arrowheads). Large arrowheads indicate cells magnified in the insets. DPI treatment (10 µM) results in punctate localization within subregions of the nucleus. Scale bar: 100 µm. (D,E) Point mutant (C179A) shows nuclear localization without H_2_O_2_ (D) and with H_2_O_2_ (0.1 µM) (E). Scale bar: 100 µm. Arrowheads, indicate transfected cells; large arrowheads indicated cell magnified in the insets. (F) Cell movement and directionality (arrows) tracked over time. Control (untreated) cells migrate slightly faster than cells treated with high H_2_O_2_ (100 µM) concentrations, following DPI treatment and when transfected with C179A-Ikk. Transfection with FL-Ikk and treatment with low H_2_O_2_ (0.1 µM) concentrations promotes migration. Scale bar: 40 µm. Ave, average, mean; LZ, leucine zipper; HLH, helix-loop-helix; NBD, Nemo-binding domain. Data are mean±s.d. ANOVA test and Tukey's multiple comparisons test were used for statistical analyses.

To assess IKKα sulfenylation, we utilized DYn-2, a chemically modified dimedone, which through click-chemistry allows for biotinylation and isolation via biotin–streptavidin interactions (Paulsen et al., 2012; Truong, 2012a) (Fig. 4A). Treatment for 5 min with H_2_O_2_ at 10 µM, a concentration that promotes cell migration, increased proteome-wide sulfenylation when assessed with streptavidin-tagged horseradish peroxidase (HRP) in western blots (Fig. 5A, upper panel). The isolated sulfenylated fraction was further probed for IKKα, showing a 19-fold increase in sulfenylated IKKα after treatment with H_2_O_2_ compared to basal (untreated) levels that presumably contained endogenously oxidized and unoxidized IKKα (Fig. 5A, lower panel). GAPDH served as positive control due to its known function as an oxidoreductase (Truong and Carroll, 2012b), which was oxidized under both basal and H_2_O_2_ conditions (the latter showing a 2-fold increase). These results demonstrate that H_2_O_2_ directly oxidizes IKKα.

We next asked whether cysteine oxidation of IKKα is required for keratinocyte migration. We had already generated two Ikkα–tdTomato reporter constructs (wild type and mutated cysteine) using zebrafish Ikk1 (encoded by *chuk*), and therefore assessed these constructs in HEK001 cells. The Ikk1 sequence is largely conserved within the kinase domain when compared with human IKKα (Fig. 5B). Following transfection into HEK001, we monitored the localization of both constructs. The wild-type (full-length) form localized to the cytoplasm and became largely perinuclear following H_2_O_2_ treatment, whereas DPI treatment led to subnuclear staining (Fig. 5C). This is consistent with the cytoplasmic and nuclear localization of human IKKα under these treatment conditions when detected using antibody staining (Fig. 3). Intriguingly, cysteine-mutated Ikk1 localized to the nucleus with and without the presence of H_2_O_2_ (Fig. 5D,E), consistent with the idea that H_2_O_2_-dependent oxidation promotes cytoplasmic localization of IKKα, which is abrogated when the kinase-specific cysteine residue is mutated. To further determine the role of IKKα oxidation in migration, we monitored the motility of transfected cells over time. Because transient transfections precluded the formation of confluent monolayers and thus scratch assays, we performed assays with unscratched cells at ∼60-70% confluence. To validate this approach, we treated some wells with 0.1 and 100 µM H_2_O_2_ and compared HEK001 migration under these conditions. This showed a migration increase after low- but not high-level H_2_O_2_ treatment, which was also seen in scratched cells (Fig. 1). Comparisons of Ikk1-transfected cells showed that the average migration distance was highest in keratinocytes that had been transfected with full-length Ikk1–tdTomato in the presence of low H_2_O_2_ concentrations (Fig. 5F). Keratinocytes that had been transfected with cysteine-mutated Ikk1 (C179A) tagged with tdTomato migrated, however, in a manner similar to that of keratinocytes treated with high concentrations of H_2_O_2_ (100 µM), or DPI. Untreated control cells traveled intermediate distances, possibly due to effects related to reduced confluence (Fig. 5F). These results suggest that IKKα cysteine oxidation and cytoplasmic localization is required for cell migration, and they further indicate that IKKα may serve as an innate surveilling protein of the redox environment to regulate keratinocyte migration and differentiation.

### IKKα oxidation promotes de-repression of *EGF* promoter activity

**Fig. 6.**
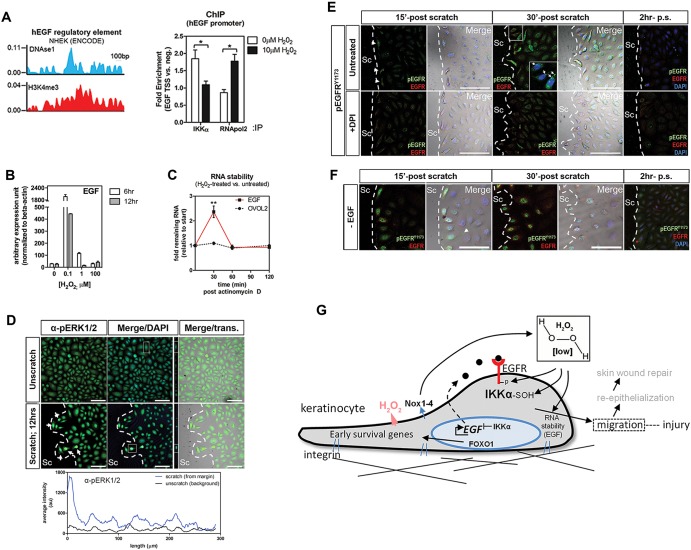
**Oxidized IKKα increases EGF expression and EGFR activity.** (A) ChIP assay in HEK001 cells with or without H_2_O_2_ shows decreased IKKα binding to the *EGF* promoter following H_2_O_2_ treatment, whereas RNA polymerase II (RNApol2) binding increases, indicative of increased *EGF* expression (two biological replicates, three technical replicates each). IP, immunoprecipitation; TSS, transcriptional start site; neg., negative control. (B) Transcriptional activation of *EGF* after H_2_O_2_ treatment. (C) H_2_O_2_ treatment (0.1 µM) increases *EGF* mRNA stability within 30 min and leads to subsequent destabilization. H_2_O_2_ does not induce ovo-like zinc finger 2 (*OVOL2*) transcription in HEK001 cells, which is known to be oxidation independent. (D) Increased ERK1/2 phosphorylation (pERK1/2) in HEK001 cells adjacent to the scratch margin and quantification of ERK1/2 phosphorylation. Trans., transmission. (E) H_2_O_2_-dependent phosphorylation of EGFR at residue Y1173 (pEGFR^Y1173^) after acute scratch injury of HEK001 cells. Intracellular recycling of EGFR phosphorylated at Y1173 in scratch margin keratinocytes (arrows, inset) shown by immunofluorescence studies. (F) Rapid phosphorylation of EGFR at residue Y1173 at the scratch margin in immunofluorescence studies in the absence of exogenous EGF. Immunostaining was also prevalent at the cell boundary (arrowhead). p.s., post scratch; 15ʹ, 15 min. (G) Wound keratinocyte migration model. High levels of H_2_O_2_ may outcompete available antioxidant complexes and lead to non-specific oxidation and apoptosis, whereas low levels stimulate cysteine sulfenylation and migration. Sulfenylation of IKKα activates keratinocyte migration through IKKα-mediated regulation of *EGF* transcription and stability. Low H_2_O_2_ levels also promote EGFR phosphorylation at Y1173. FOXO1 might regulate H_2_O_2_ levels to promote migration. Scale bars: 100 µm. One-way ANOVA and Tukey's multiple comparison post hoc tests were utilized. Significance: *P<0.05, **P<0.01. Sc, scratch.

Evidence so far suggests an important function for oxidized IKKα in keratinocyte migration. How this translates into downstream functions that promote migration is unclear. This could be mediated, in part, by the cis-repressive transcriptional binding activity of IKKα to the *EGF* promoter region (Sil et al., 2004), which regulates autocrine loops within keratinocytes (Liu et al., 2008). To assess the role of oxidized IKKα in *EGF* promoter activity, we performed chromatin immunoprecipitation (ChIP) using H_2_O_2_-treated keratinocytes. Using ENCODE and genome assembly for human coordinates (GRCh38; GenBank assembly accession GCA_000001405.15), we designed primers to target the predicted *EGF* promoter region in which IKKα may bind (Fig. 6A, left panel). ChIP results showed that endogenous IKKα bound to the *EGF* promoter region under unstimulated (no H_2_O_2_ treatment) conditions (Fig. 6A, right panel). In contrast, binding was reduced upon treatment with 10 µM H_2_O_2_, consistent with the idea that oxidation promotes EGF promoter activity by reducing IKKα nuclear localization. RNA polymerase II, a general marker for precursor RNA synthesis, showed reciprocal binding activity at the *EGF* promoter region. Consistent with these findings, siRNA knockdown of IKKα led to a >1.6-fold increase specifically in *EGF* transcripts, whereas IKKβ and IKKγ (*IKBKG*) transcripts remained unaffected (Fig. S4E), validating a functional relationship between IKKα and EGF. Similarly, we observed highly elevated *EGF* transcript levels upon treatment with low exogenous H_2_O_2_ (0.1 µM) concentrations but, intriguingly, not when cells were treated with high concentrations (100 µM) (Fig. 6B).

Since growth factor levels are regulated through rapid decay of their transcripts (Tang et al., 1997), we also investigated the potential role of H_2_O_2_ on *EGF* mRNA stability. Following pre-treatment with actinomycin D for 1 h, which produced steady-state *EGF* mRNA levels (Fig. 6C, 0 min time point), we observed within 30 min a transient increase and subsequent degradation of *EGF* mRNA at low H_2_O_2_ concentrations but not in the absence of H_2_O_2_. We also did not see this effect when assessing a known H_2_O_2_-independent control transcript, *OVOL2* (Marinari et al., 2008) (Fig. 6C). This rapid effect of H_2_O_2_-dependent *EGF* mRNA accumulation may occur through the activation of non-genomic upstream signaling regulators – i.e. ERK1 and ERK2 (ERK1/2; also known as MAPK3 and MAPK1, respectively) (Nagashima et al., 2014), which we also observed to be upregulated at the scratch margin (Fig. 6D).

Our findings suggest that oxidized IKKα regulates *EGF* promoter activity, which is likely to regulate EGFR signaling given the activation of its downstream effector ERK1/2 at the scratch wound. However, EGFR could also be additionally activated by other mechanisms, such as oxidation in an EGF-dependent manner, as shown previously in squamous carcinoma cells (Paulsen et al., 2012). We therefore investigated H_2_O_2_-dependent EGFR activation using immunofluorescence staining and western blot analysis. This showed rapid transient phosphorylation of EGFR at residue Y1173 and internalization at the keratinocyte scratch margin, which dissipated under DPI treatment (Fig. 6E). We also assessed another previously shown EGFR phosphorylation site (Y1068) in squamous carcinoma cells (Paulsen et al., 2012), which did not show a difference in phosphorylation with or without H_2_O_2_ treatment (data not shown). Phosphorylation of EGFR at Y1173 also occurred under EGF withdrawal, suggesting wound-dependent autophosphorylation that could be mediated by endogenous EGF and/or H_2_O_2_ production (Fig. 6F). Quantitative western blot analyses revealed a ∼3-fold increase in phosphorylation of EGFR at Y1173 (normalized to total EGFR) after 1 h of treatment with low H_2_O_2_ concentrations (0.1 µM), and a ∼28-fold decrease with DPI (Fig. S4H). These findings indicate that EGFR activity in HEK001 cells is regulated by H_2_O_2_.

## DISCUSSION

Long-term HEK001 cultures (i.e. 14 DIV) resulted in deregulated proliferative (PCNA) and basal markers (KRT14) in the presence of high Ca^2+^, as observed for other keratinocyte cell lines previously (Micallef et al., 2009). This hyperproliferation may be due to Ca^2+^ resistance, response to hypotonic stress or a lack of differentiation factors besides Ca^2+^ in the medium. Furthermore, it is possible that these differences also reflect heterogeneous culture preparations, as it is unclear whether HEK001 is a pure keratinocyte cell line. Since our studies were performed for relatively short time periods, these late changes should not have influenced our findings. We further show that similar to human lung epithelial cells, primary epithelial cells produce scratch-induced H_2_O_2_ and show increased migration upon H_2_O_2_ treatment, similar to HEK001 cells. Thus, our findings are also likely to be relevant to other epithelial cells types and to the *in vivo* context.

We assessed enzymes that are possibly required for H_2_O_2_ production given various reports in the literature. For instance, *Drosophila* and zebrafish embryos, and human lung and neonatal foreskin epithelial cells produce H_2_O_2_ via DUOX (Choi et al., 2014; Niethammer et al., 2009; Razzell et al., 2013; Wesley et al., 2007). NOX1 and NOX4, and the common subunit P22PHOX (encoded by *CYBA*) are, in contrast, activated in cancer cells (Ha et al., 2016; Ito et al., 2016) and have been associated with keratinocyte-specific activity (Chamulitrat et al., 2004; Nam et al., 2010; Sun et al., 2016). While DUOX1 and NOX1 expression remained unchanged during scratch wound repair, NOX4 expression was significantly upregulated, although relatively late – after 12 h. It is possible that the mRNA expression data may not fully reflect protein expression levels within cells since alternative regulatory mechanisms could be at play. For instance, regulation of subunits that stabilize Nox enzymes or an increase in RNA and/or protein stability may be sufficient to generate H_2_O_2_ over the course of multiple hours. At present, however, our studies do not permit conclusions about NOX4 involvement given that the expression kinetics of NOX4 do not match the expected H_2_O_2_ production profile after scratch wounding.

Our findings show that IKKα is an oxidation target of H_2_O_2_. IKKα is mostly known for its kinase-dependent regulation of NF-κB signaling, such as during cellular responses to stress (Israel, 2010). In the differentiated epidermis however, IKKα has additional kinase- and NF-κB-independent nuclear repressor functions (Fukazawa et al., 2010) to maintain skin homeostasis (Descargues et al., 2008; Hu et al., 2001; Liu et al., 2008). Our results are in line with these findings, suggesting that NF-κB independent mechanisms are involved in oxidation of IKKα, leading to keratinocyte migration. Previous studies have shown that the nuclear function of IKKα is associated with production of an unidentified soluble factor termed keratinocyte differentiation-inducing factor (kDIF), which can promote terminal differentiation (Descargues et al., 2008; Hu et al., 2001). In parallel, nuclear IKKα is also known to promote keratinocyte differentiation through cis-repression of *Egf* in mice (Liu et al., 2008). Our study, for the first time, demonstrates that the *EGF* repressive activity can be blocked by H_2_O_2_ through promotion of IKKα cytoplasmic accumulation that stimulates keratinocyte migration. We observed increased IKKα at localized subcellular regions of keratinocytes at the scratch margin. At this point, it remains to be shown how IKKα is regulated by H_2_O_2_. For instance, a steady-state flow of IKKα between the cytoplasm and nucleus might be present under homeostatic conditions, and oxidation of cytoplasmic IKKα may lead to cytoplasmic accumulation and reduced nuclear flow. Alternatively, nuclear IKKα might be oxidized, leading to active nuclear export. Indeed, it has been shown previously that H_2_O_2_ can be detected in the nucleus of mammalian cells and in whole organisms, such as *Caenorhabditis elegans* (Dickinson et al., 2011). Cytoplasmic sequestration of IKKα indicates alternative molecular functions that are independent of NF-κB signaling. It is known that cysteine oxidation can change protein conformation and enzymatic activity. Hence, in addition to dissociation from the *EGF* promoter, H_2_O_2_-modified IKKα may regulate signaling pathways using its kinase function, which would be in line with the observation that IKKα enzymatic inhibition impaired scratch closure, yet endogenous NF-κB inhibition did not. It is possible that oxidized IKKα actively sequesters NF-κB in the cytoplasm, which remains to be investigated. Alternatively, oxidized IKKα could potentially regulate other migration-promoting target genes through cytoplasmic sequestration of nuclear co-repressors (Fernández-Majada et al., 2007).

Our data shows that low but not high exogenous H_2_O_2_ concentrations induce *EGF* mRNA expression, correlating with the cytoplasmic accumulation of IKKα and the induction of migration. Our findings further suggest a concentration-related regulation of IKKα sulfenylation, which could be mediated by the availability of intermediate enzymes that control for the sulfenylation reaction. This would be in line with our finding that strongest sulfenylation was observed about 2 h after scratch wounding, despite H_2_O_2_ being produced as early as ∼30 min after wounding. Thus, this suggests that initially there is IKKα-independent EGFR signaling, which is consistent with the rapid transient H_2_O_2_-dependent activation of EGFR via phosphorylation of residue Y1173, and the temporal stabilization of *EGF* messenger RNA by H_2_O_2_. The stabilization could be achieved through lack of degradation of the messenger RNA. Based on these findings, we propose a model in which, initially, EGFR signaling is activated by direct oxidation, whereas oxidation of IKKα subsequently induces EGF expression through activation of the *EGF* promoter. This might ultimately lead to sustained EGFR signaling that is required for persistent migratory activity of keratinocytes (Fig. 6G). Further studies are required to determine the relationship between IKKα oxidation and EGFR activity, and the role of EGFR oxidation in basal keratinocyte migration. It is known that EGFR is overly active in many cancers and that IKKα genetic/epigenetic downregulation (Maeda et al., 2007; Park et al., 2007) and cytoplasmic sequestration (Marinari et al., 2008) can trigger oncogenic pathways, of which the regulators of sequestration remain unknown. Interestingly, to our knowledge, loss of IKKα is associated with hyperproliferation but not tumor metastases. By contrast, IKKα expression itself is known to promote a metastatic phenotype in some tumors (Luo et al., 2007). Thus, our findings may have even broader implications, as elevated ROS levels exist in tumors (Liou and Storz, 2010) and low but not high H_2_O_2_ levels may act directly on IKKα in the cytoplasm to regulate metastasis.

## MATERIALS AND METHODS

### Cell lines, differentiation assay, H_2_O_2_ treatment, scratch wound and inhibition assays

HPV16-transformed human epidermal keratinocytes [HEK001; American Type Culture Collection (ATCC), CRL-2404] were maintained in keratinocyte-serum free (KSF) medium (Gibco-Brl 17005-042) supplemented with 5 ng/ml human recombinant EGF, low CaCl_2_ (0.06 mM) and 2 mM L-glutamine (without bovine pituitary extract). Cells were incubated under 5% CO_2_ and a 92% humidified atmosphere at 34°C, and seeded (4×10^4^ cells/cm^2^) in tissue culture plates pre-coated with type I collagen (Gibco-Brl, R-011-K).

For RNA and scratch analyses, cells were cultured in 12-well plates (Corning, 3513) and 8-chamber glass-bottom dishes (In Vitro Scientific, C8-1.5H-N), respectively. The culture medium was changed every 2-3 days and the dishes were confluent by 2 DIV post-seeding.

Keratinocyte differentiation was initiated with 2 mM CaCl_2_ and monitored for ∼2 weeks. The scratch assay was used to evaluate cell migration and wound recovery (Goetsch KP, Niesler CU, 2011). Cells were grown to confluence, replaced with EGF-lacking medium for 12 h, refreshed with complete medium, and glass Pasteur pipettes were used to make vertical scratches along the surface of the vessels. Wells were immediately washed with PBS to avoid re-attachment of disassociated cells.

Cells were treated with H_2_O_2_ and inhibitors as indicated in the text and in Materials and Methods under ‘Antibodies and chemical inhibitors’. For western blots, after H_2_O_2_ treatment, cells were lysed in RIPA buffer supplemented with phosphatase and proteinase inhibitors, and analyzed using the NuPage system (Invitrogen). For RNA expression analyses, wounds were generated using a comb-like device, and RNA was subsequently purified. For EGFR immunofluorescence studies (see antibody staining), cells were treated without EGF for >12 h and then scratched followed by supplementation with complete medium. For some assays, cells were pre-treated for 1 h with DPI to eliminate endogenous ROS.

Primary epidermal keratinocytes (ATCC; PCS-200-010) were grown in KSF medium, and scratch assays and treatment with H_2_O_2_ were performed according to HEK001 cell protocols.

### Intracellular H_2_O_2_ detection, cell viability

HEK001 were pre-treated for 30 min with the H_2_O_2_ sensor HPF (Cayman Chemical, CAS: 728912-45-6), scratched and then detected by confocal imaging. HPF was maintained as a 1 mM stock in DMSO. Initially, the optimal signal:noise ratios were empirically determined using 0.1, 1, 4 and 10 µM concentrations. Each condition was tested in four separate wells with four replicate images/well. Cell viability and apoptosis were monitored using the 6-CFDA kit (ThermoFisher) and Annexin-V–Cy3 kit (APOAC, Sigma).

### Keratinocyte Boyden chamber migration assay

Assays were performed using Millicell with hanging Transwell polyethylene terephthalate inserts (8 μm pore size, Millipore, PIEP12R48) for impedance-based detection of migrated cells. 200 µl of serum-free medium that contained 5×10^4^ 12 h-unstimulated cells was added to the upper compartment, while the lower compartment was filled with 750 µl KSF medium with or without EGF, H_2_O_2_ or inhibitors. After 12-24 h, non-migratory cells remaining in the upper chamber were removed with cotton swabs. Migratory cells on the lower surface of the Transwell membrane were fixed (4% PFA) and stained with Giemsa/DAPI. Cells were imaged on an FV1000-confocal microscope (Olympus) and counted using ImageJ.

For mitomycin C experiments, a stock solution of mitomycin C (Sigma, M0503) was made fresh in sterile water. A working concentration of 10 µg/ml mitomycin C was determined by performing bioactivity assays. For migration assays, cells were co-cultured using Boyden Transwell chambers along with mitomycin C and H_2_O_2_, and the number of migrating cells post 12 h were quantified using DAPI staining.

### Micromass assay

HEK001 cells were expanded in T-25 flasks, trypsinized and resuspended in 1 ml of complete medium. Cells were transferred to 1.5 ml tubes and pelleted by centrifugation at 0.2 ***g***. The medium was aspirated and cells resuspended in KSF-medium to perform cell counts. Cells were resuspended in a final volume [=number of wells×volume per well (in 24-well plates)] where the seeding volume was 30 µl per well at 0.5×10^6^ cells. Cells were placed at the center of each well, carefully placed into a CO_2_ incubator and allowed to attach for 2-4 h. 1 ml of KSF+EGF was carefully added to each well. The medium was replaced every other day without disturbing the centered micromass. The H_2_O_2_ detection assay was performed after 4 days in culture.

### qPCR and *EGF* mRNA stability assay

Total RNA was purified using the RNeasy mini kit (Qiagen). RNA was reversed-transcribed using Superscript III reverse transcriptase (Invitrogen) and equal amounts of poly-dT and random hexamer oligonucleotides. Gene expression was normalized to human *ACTB* mRNA and analyzed using the comparative CT Livak method (Livak and Schmittgen, 2001) using BrilliantII SYBR^®^ Green qPCR Master Mix (Agilent) (see Table S1 for primer sequences).

For *EGF* mRNA stability assessment, cells were pre-treated with actinomycin D (2.5 µg/ml; Sigma-Aldrich) for 1 h, followed by treatment with 0.1 µM H_2_O_2_ before RNA extraction. Both untreated and H_2_O_2_-treated samples with actinomycin were compared to the starting samples on graphs.

### Antibodies and chemical inhibitors

Antibodies against human ERK1 (phosphorylated at T202 and Y204) and ERK2 (phosphorylated at T185 and 187) (Abcam, ab50011), EGFR (phosphorylated at Y1068; Abcam, ab32430), EGFR (ThermoFisher, MA5-12880), EGFR (phosphorylated at Y1173; Abcam, ab32578), sulfenic acid modified cysteine/2-thiodimedone (Millipore, AB330), forkhead box O1 (LSBio, LS-C123562), IKKα (Abcam, ab4111), NF-κB (p52 and p100 subunits) (ThermoFisher, PA5-27340) and NF-κB (p50 and p105 subunits) (ThermoFisher, PA1-14284) were used at a 1:200 dilution for immunofluorescence studies. AlexaFluor488-conjugated anti-rabbit IgG (Molecular Probes, Invitrogen) and Cy3-conjugated anti-mouse IgG (Millipore) were used as secondary antibodies at a 1:1000 dilution. Inhibitors were kept as stock solutions in DMSO for FOXO1 (Millipore, 344355; IC_50_=33 nM), DPI (Sigma-Aldrich, 2926), NF-κB (Santa Cruz Biotechnology, JSH-23; IC_50_=7.1 μM), IKK Wedelolactone (Millipore, 401474; IC_50_=10 μM), EGFR (Millipore, 324673; IC_50_=20 nM) and apocynin (Santa Cruz Biotech, sc-203321; IC_50_=10 μM). DYn-2 was a kind gift from Kate Carroll (Scripps Institute).

### Antibody, Crystal Violet and phalloidin staining

HEK001 cells were fixed in 4% PFA (15 min) and permeabilized in 0.25% Triton X100 (10 min) at room temperature. Cells were blocked with 1%BSA with 10% goat serum and 0.1% Tween-20 in PBS (30 min) and incubated with primary antibodies (1:200) in blocking buffer overnight at 4°C. Cells were washed and labeled with appropriate secondary antibody (1:1000) and DAPI or Hoechst 33342 (1:5000). To detect sulfenylated proteins, keratinocytes were incubated for 2 h in DMSO or 2 mM dimedone (5,5-dimethyl-1,3-cyclohexanedione) (Cayman Chemical), scratch wounded or treated with H_2_O_2_ and further incubated for 2 h. Samples were fixed and stained with a polyclonal anti-dimedone antibody (EMD Millipore, 07-2139). The cells were washed and stained with anti-rabbit AlexaFluor488-conjugated secondary antibody (Life Technologies) for detection on a confocal microscope. To stain 7-DIV cells that have been differentiated with 2 mM Ca^2+^, cells were incubated in 0.2% Crystal Violet with 10% ethanol for 10 min, followed by imaging on an inverted stereomicroscope (Zeiss, Germany). Phalloidin–AlexaFluor647 (ThermoFisher) was used to assess the actin cytoskeleton. Cells were grown to confluency and either left untreated or treated with 0.1 and 1 µM H_2_O_2_ for 2 h. Cells were fixed in 4% PFA (15 min) and washed in PBS. Phalloidin staining was performed according to the manufacturer's protocol. The cells were washed in PBS and immediately imaged on an FV1000 confocal microscope (Olympus).

### Azide labeling and click-chemistry biotinylation of labeled oxidized proteins

The alkyne probe DYn-2 [4-(pent-4-yn-1-yl)cyclohexane-1,3-dione; 5 mM] (gift from Kate Carroll, The Scripps Institute, San Diego, CA, and purchased from Cayman Chemical, 11220) was added to HEK001 cells for 1 h at 37°C. The cells were lysed in modified RIPA (200 U/ml catalase, EDTA-free protease and phosphatase inhibitors) and pre-cleared of endogenous biotinylated proteins using pre-equilibrated NeutrAvidin resin (Thermo, 29202). Probe-labeled proteins were detected using click-chemistry (Invitrogen, C10276) with solubilized 4 mM biotin azide (Invitrogen, B10184). Proteins were precipitated using methanol and electrophoresed. For whole proteome detection, the membrane was blotted with streptavidin–HRP and loading antibodies [Santa Cruz Biotech, IKKα (B-8), sc-7606; GeneTex, GAPDH, 124503]. For detection of specific oxidized proteins, biotin-azide-labeled proteins were immunoprecipitated using NeutrAvidin agarose beads, eluted with 8 M guanidine-HCL, dialyzed using a Slide-A-Lyzer^®^ MINI dialysis device (Thermo Scientific; 88401), concentrated using Amicon Ultra(3 K) centrifugal filters (Millipore, 500324) and re-probed for western analyses.

### Silencing of human IKKα expression using an RNA interference assay

A 50 µM stock of Silencer^®^ Select validated siRNA oligonucleotides against human IKKα (Locus ID: 1147) (Ambion^®^, Life Technologies; siRNA ID: s3077) was resuspended in nuclease-free water and stored at −80°C. For IKKα mRNA knockdown validation, HEK001 cells were grown to 60-70% confluence [1.6×10^5^ cells/well (12 well-plate)], and siRNAs (10-20 pmol) were transfected into cells for 24 h using the Lipofectamine^®^ RNAiMAX reagent and methodology (Life Technologies). Gene expression (qPCR) was investigated after 48 h transfection (see Table S1 for primers). Validation of protein knockdown was also performed at this time using an anti-IKKα (Abcam, ab4111) antibody in western blots. For scratch assays using siRNA-transfected HEK001, cells were trypsinized and then re-plated at full confluence in 8-well chambers at 48 h post-transfection. siRNA-transfected cells were scratched 12 h after re-plating and processed accordingly (two biological replicates performed in triplicates).

### ChIP-qPCR analyses of the human *EGF* promoter

HEK001 cells were grown to 80% confluence and cultured for 12 h without EGF. EGF was reintroduced with or without 10 µM H_2_O_2_ for 30 min. *In vivo* crosslinking of chromatin was performed with 1% formaldehyde and quenched with glycine. Digestion and isolation of chromatin-bond DNA by immunoprecipitation (ChIP) was performed using a magnetic ChIP kit (26157, Pierce) based on the manufacturer's protocol. To assess physical binding of components to genomic sequences, RNA polymerase II (included in ChIP kit) and IKKα (sc-7606, Santa Cruz Biotech), ChIP-grade antibodies were used for immunoprecipitation. Subsequent PCR quantification was performed using SYBR™ green (600882, Agilent) with primers recognizing the *EGF* promoter and intergenic negative control DNA sequences (Table S1). The current genome assembly for human coordinates (GRCh38; GenBank assembly accession GCA_000001405.15) was used to design primers. The negative control was used to normalize for DNA content to calculate the enrichment of the regulatory *EGF* region according to the Livak method.

### Generation and transient transfection of Ikk constructs

Zebrafish *Ikk1* (*Chuk*) cDNA was purchased from Open Biosystems (Clone ID: 5914247, GE Dharmacon). Full-length (FL)-*Ikk1* was amplified with Advantage 2 Taq Polymerase (Clontech) using forward primer 5′-GCTAGCTGTCAATATGGAGAAACCCCCT-3′ and reverse primer 5′-TAATGGATTTGGTACCGACAAACGCGCTGATTTA-3′. The amplified PCR products were cloned into pCR Blunt II-TOPO using the Zero Blunt^®^ TOPO^®^ PCR Cloning Kit (Life Technologies). To generate tdTomato fusion constructs, pCRTOPO-FL-Ikk1 and ptdTomato-N1 vector (Clontech) were digested with KpnI and NheI, and ligated. The Ikk1(C179A)–tdTomato variant was generated from FL-Ikk1–tdTomato using site-directed mutagenesis (GeneArt^®^ Site-Directed Mutagenesis PLUS Kit, LifeTechnologies). The constructs were subsequently cloned into a plasmid containing the zebrafish *tp63* promoter (pT2KXIG_*tp63*:AcGFP, courtesy of Gromoslav Smolen, Harvard University, Cambridge, MA). First, GFP was removed via digestion with BamHI and NotI, and both sites were Klenow blunt-ended. The Ikk1–tdTomato plasmids were digested with SnaBI and SfoI and ligated into pT2KXIG_*tp63* to generate *tp63*:FL-ikk1–tdTomato and *tp63*:ikk1(C179A)–tdTomato. HEK001 were transfected using Nanofectin (PAA, Q050-005) when 60% confluent in 8-chamber vessels. It was determined that 0.5 µg of DNA per 0.6 µl of transfection reagent was optimal.

### Confocal imaging and relative comparison, and data analyses

Laser scanning confocal microscopy images were obtained using an inverted Olympus FV1000 or Zeiss LSM510 unit. *z*-stacks obtained from 1-3 µm/slice per sample and a speed of 12.5 (µs/pixel) were projected. All other parameters (e.g. pinhole diameter, gain, laser intensities) were kept constant. The series of projected ‘*z*-axis’ images were used to calculate average fluorescent intensity profiles per channel using the line series and box analyses tools in the Fluoview software v. 4.1 (Olympus). A minimum of 16 evenly distributed ‘scratch’ lines were averaged per image, or six evenly distributed boxes were averaged per image to generate average (background) fluorescence intensities. The fluorescence intensity was never saturated (maximum 4096 intensity level) during imaging. Samples within the same well were compared; i.e. scratched regions were compared to unscratched regions, and the background was corrected between the scratch margins and averaged unscratched ‘margin-free’ regions within the same well and vicinity. The line tool was used to measure length:width ratios for most elongated sulfenylated cells (a total of ∼60 per group). Cell–cell distances and fluorescence intensities were measured in Imaris (Bitplane) software using the line tool and spots function, respectively. Migration of Ikk1-transfected HEK001 cells was documented with time-lapse imaging for 8 h using an incubation chamber.

### Statistical analyses

Statistical comparisons were made using Prism 6 software (GraphPad). As indicated in the figures, the unpaired Student's *t*-test with a 95% confidence interval was used to compare the means of two unmatched groups, assuming that the values followed a Gaussian distribution. For multiple comparison tests of three or more groups, one-way ANOVA with an α value=0.05 (95% confidence interval) and Tukey's multiple comparison post hoc tests were utilized to compare the means of each column. Significance is denoted with asterisks in the figures: *P<0.05, **P<0.01, ***P<0.001, ****P<0.0001.
